# Multimorbidity, healthcare use and catastrophic health expenditure by households in India: a cross-section analysis of self-reported morbidity from national sample survey data 2017–18

**DOI:** 10.1186/s12913-022-08509-x

**Published:** 2022-09-12

**Authors:** Anup Karan, Habib Hasan Farooqui, Suhaib Hussain, Mohammad Akhtar Hussain, Sakthivel Selvaraj, Manu Raj Mathur

**Affiliations:** 1 Indian Institute of Public Health Delhi, Public Health Foundation of India, Gurugram, 122002 India; 2 College of Medicine, QU Health, Qatar University, Doha, Qatar; 3 Barwon South West Public Health Unit, Barwon Health, Geelong, Victoria Australia; 4 Health Economics, Financing and Policy, Public Health Foundation of India, Gurugram, 122002 India; 5 Institute of Dentistry, Bart’s and The London School of Medicine and Dentistry, New Road, London, E1 2AT UK

**Keywords:** India, multimorbidity, out-of-pocket expenditure, catastrophic expenditure

## Abstract

**Background:**

The purpose of this research is to generate new evidence on the economic consequences of multimorbidity on households in terms of out-of-pocket (OOP) expenditures and their implications for catastrophic OOP expenditure.

**Methods:**

We analyzed Social Consumption Health data from National Sample Survey Organization (NSSO) 75th round conducted in the year 2017–2018 in India. The sample included 1,13,823 households (64,552 rural and 49,271 urban) through a multistage stratified random sampling process. Prevalence of multimorbidity and related OOP expenditure were estimated. Using Coarsened Exact Matching (CEM) we estimated the mean OOP expenditure for individuals reporting multimorbidity and single morbidity for each episode of outpatient visits and hospital admission. We also estimated implications in terms of catastrophic OOP expenditure for households.

**Results:**

Results suggest that outpatient OOP expenditure is invariably lower in the presence of multimorbidity as compared with single conditions of the selected Non-Communicable Diseases(NCDs) (overall, INR 720 [USD 11.3] for multimorbidity vs. INR 880 [USD 14.8] for single). In the case of hospitalization, the OOP expenditures were mostly higher for the same NCD conditions in the presence of multimorbidity as compared with single conditions, except for cancers and cardiovascular diseases. For cancers and cardiovascular, OOP expenditures in the presence of multimorbidity were lower by 39% and 14% respectively). Furthermore, around 46.7% (46.674—46.676) households reported incurring catastrophic spending (10% threshold) because of any NCD in the standalone disease scenario which rose to 63.3% (63.359–63.361) under the multimorbidity scenario. The catastrophic implications of cancer among individual diseases was the highest.

**Conclusions:**

Multimorbidity leads to high and catastrophic OOP payments by households and treatment of high expenditure diseases like cancers and cardiovascular are under-financed by households in the presence of competing multimorbidity conditions. Multimorbidity should be considered as an integrated treatment strategy under the existing financial risk protection measures (*Ayushman Bharat*) to reduce the burden of household OOP expenditure at the country level.

**Supplementary Information:**

The online version contains supplementary material available at 10.1186/s12913-022-08509-x.

## Introduction

Globally, an unprecedented increase in non-communicable disease (NCD) risk factors [1] has led to a rise in chronic health conditions [2]. These NCDs interact and tend to cluster together leading to a state of multimorbidity—defined as the co-existence of two or more NCDs in an individual, without a defining primary index disease [3]. Multimorbidity becomes progressively more common with ageing and is linked to high mortality and reduced functional status [4]. Multimorbidity is challenging not only to the patient, because of negative health consequences, but also to the healthcare system due to multiple consultations for care [5, 6].

A large retrospective study from England has reported that depending upon the number of NCDs and the age group of participants included, the prevalence of multimorbidity is highly variable. The study suggests that patients with multimorbidity accounted for 52.9% of general practice consultations, 78.7% of prescriptions, and 56.1% of hospital admissions [7].

The situation in the low- and middle-income countries is no different. A systematic review reported that in South Asia, the prevalence of multimorbidity varied between 4.5% to 83%. The review also highlighted lowered physical functioning and increased healthcare utilization as the most frequently reported outcomes [8]. Furthermore, in developing countries where healthcare is overwhelmingly financed by out-of-pocket (OOP) payments, households face a significant drain on their resources. The latest evidence from India suggests that an estimated 55 million households experienced catastrophic healthcare expenditure in the year 2014 and non-communicable disease constitutes a significantly large proportion of the total catastrophic expenditure [9].

While a consensus methodology for measurement of economic consequences of multimorbidity is yet to be established, literature on treatment cost of multimorbidity [10, 11] has increased. A systematic review of the cost of illness (COI) studies on multimorbidity concluded that despite substantial methodological variations between COI studies across different countries and health system settings, there is consistent evidence of considerable economic burden associated with multimorbidity [12]. Past research has also demonstrated that households incur high OOP expenditure for NCD treatment [10, 13–17].

However, there is little evidence of whether households can adequately finance the healthcare needs of each member of the household for each NCD in the presence of multimorbidity. In this study, using nationally representative data from India, we estimated households’ OOP expenditure for selected NCDs when reported as a standalone disease and for the same NCD in presence of competing NCDs (multimorbidity).

We argue that in the presence of competing NCDs (multimorbidity), households fail to finance treatment and care of all the NCDs adequately that involve large healthcare expenditure. The competing risk and demand for care for each NCD may force households to forego or underfinance treatment and care of co-existing morbidities. In general, health conditions such as cancers, injuries, and cardiovascular diseases are known to involve high healthcare expenditure [9] and in presence of competing NCDs (multimorbidity), OOP expenditures may turn catastrophic for the households.

Our main objective is to compare households’ OOP expenditure for selected NCDs when reported as single morbidity versus OOP expenditure on the same NCDs in presence of competing NCDs (multimorbidity). In addition, we also aim to report the implications in terms of catastrophic OOP expenditure of selected NCDs on the households.

## Material and methods

### Data source and sample

We analysed the Social Consumption: Health (SCH) data from National Sample Survey Office (NSSO) 75^th^ Round (July 2017 to June 2018) [18]. Nationally, ~ 1,13,823 households (64,552 in rural areas and 49,271 from urban areas) were included in the survey through a multistage stratified random sampling process. The information is collected from sample households using a questionnaire schedule (25.0). The SCH (2017–18) survey provides disease-level information on healthcare use and OOP expenditures in outpatient and inpatient care separately. The survey also collected information on the self-reported morbidity status of individual household members in addition to a range of socio-economic identifiers.

The NSSO schedule recorded the response of individuals/households to specific questions eliciting information on healthcare utilization and the reason for the same. For example, to determine any chronic conditions, respondents were asked a screening question “whether suffering from any chronic/other ailments?” (yes/no), with a recall period of 15 days and “whether hospitalised?” (yes/no), with a recall period of 365 days. We used these two questions to estimate morbidity burden and hospitalisation rates in our study. Once responded “yes” against anyone or both of these two questions by participants, follow-up questions like nature of ailment (“What was the nature of the ailment”?) nature of treatment (allopathic, indigenous, etc.), expenditure on treatment (disaggregated by 5–6 items of expenditure) and other details related to the utilization of healthcare services (such as public, private, charity trust etc.) were asked. The survey used a common set of nature of ailment in both the recall period references.

The survey asked about the nature of the ailment, classified by 60 different health conditions both for inpatient (365 days reference period) and outpatient (15 days reference) treatment. From these health conditions, non-communicable diseases and comorbidities for each individual can be identified. Respondents also recorded more than one condition, if they suffered during the respective recall periods. We matched the disease condition in the surveys to broad ICD disease classification to distinguish between major non-communicable diseases (including injuries) and communicable diseases [13]. The SCH survey also separately records expenses incurred for inpatient and outpatient care with respective reference periods for each episode of treatment (see Additional file 1: Appendix Table-I).

### Definition of multimorbidity

We considered an individual as having multimorbidity if (i) currently living with two or more non-communicable diseases [3]; or (ii) hospitalized due to multimorbidity in the year preceding the survey, whether or not the affected individual was currently alive.

### Outcome measures

We report (a) prevalence of multimorbidity by gender and age groups, (b) prevalence of selected non-communicable diseases, (c) mean OOP expenditure on outpatient care per episode for selected NCDs in the preceding 15 days, (d) mean OOP spending per hospitalization for selected NCDs in the preceding year and (e) proportion of households with selected NCDs related expenditures (outpatient and/or hospitalization) reporting catastrophic expenditure [18, 19] (at two thresholds: OOP being more than 10% and alternatively 20% of households’ total consumption expenditure). All prevalence and mean expenditure outcomes were estimated and reported for ages 40 years and above after applying sampling weights available in the data.

### Statistical analysis

Prevalence of NCDs was standardized to the age and sex distribution of the Indian population for the year 2017–18. We prepared a matrix of all NCDs reported in the NSSO survey and reported the proportion of individuals with no NCD, single NCD and co-existent NCDs among all individuals in the survey population.

We estimated the cost of treatment of selected NCD by estimating the mean OOP expenditure for each episode of outpatient visit and hospital admission. Mean OOP expenditure was estimated for the selected NCD in case of single morbidity as well as multimorbidity. For example, we estimated expenditure on cancer treatment when an individual had cancer alone versus expenditure on cancer when an individual had another NCD in addition to cancer.

To estimate the burden of OOP expenditure on the treatment of NCDs at the household level, we estimated mean per capita OOP expenditure, OOP expenditure as a share of total household consumption expenditure and the catastrophic nature of OOP expenditure as defined by the percentage of households reporting OOP payments being higher to certain thresholds (for example 10% or 20%) of households’ total consumption expenditure, among the households reporting NCDs. We also used households’ non-food expenditure as an alternative to total consumption expenditure [19, 20] for estimating the share of OOP expenditure and catastrophic OOP expenditure.

Since NCDs may be associated with a large number of socio-economic and demographic confounders [21], a direct comparison of individuals reporting a single NCD with those reporting more than one NCD may produce a biased result. To address this, we created a matched sample of individuals with single and multimorbidity by controlling a range of observed socio-economic and demographic indicators and estimated the difference in OOP expenditure for treatment of any particular NCD across individuals with and without multimorbidity in the matched sample.

We used Coarsened Exact Matching (CEM) method to create a sub-sample of individuals reporting 2 or more NCDs (‘treatment’ group) and a single NCD (‘control’) with minimum possible differences in the observed socio-economic and demographic indicators. The CEM method has been used in previous studies for estimating the economic burden of chronic disease [22, 23]. CEM uses the principle of Exact Matching (EM) and balances each indicator used in the matching by coarsening each variable into groups. The advantage of using CEM, as against other popular matching methods such as Propensity Score Matching (PSM), is that CEM guarantees a reduction in biases in each indicator as compared with the ex-ante situation [23]. A weighting variable generated by the CEM method is used to equalize the number of observations within comparison groups [24]. The variables used for CEM in the present analysis included: (i) geographic region (6 categories), (ii) area of residence (rural–urban), (iii) monthly per person consumption expenditure (10 categories), (iv) religion (Hindu, Minority), (v) caste (3 categories), (vi) main source of livelihood (4 categories), (vii) age groups (4 categories), (viii) level of education (3 categories), (ix) safe latrine (dichotomous), (x) safe water (dichotomous) (xi) safe garbage disposal (dichotomous), (xii) safe energy (dichotomous), and (xiii) health insurance (insured, not-insured) (Additional file 1: Appendix Table A-IV for categorization and sample size).

CEM reduced the sample size from 37,287 to 37,202 (from 31,240 to 30,479 in the control group and from 6,747 to 6,723 in the treatment group). The balancing results reflect that although there is a small change in the overall imbalance, many covariates reflect a significant reduction in the biases in the matched sample (Additional file 1: Appendix Table A-V). The multivariate L1 imbalance after matching reduced from 0.964 to 0.956, whereas among the individual covariates, bias reduced by 93% for a geographical region, 29% for monthly per capita expenditure, 67% for household type and 41% in the case of insurance.

As a part of sensitivity analysis, we also used the PSM method to create a matched sample. (Additional file 1: Appendix Table A-VI) In addition, we re-estimated our results after excluding households that experienced a death in the previous year. Data were analyzed using Stata software V.15.0 (StataCorp LP, College Station, Texas, USA) and *p* values of less than 0.05 were considered statistically significant. All analyses were carried out using sampling weight.

### Ethical approval and consent to participate

The study uses anonymized secondary data which is publicly available from the NSSO and hence doesn’t involve any ethical issues and approval from an ethics committee or consent to participate.

## Results

### Prevalence of NCD

**Fig. 1:**
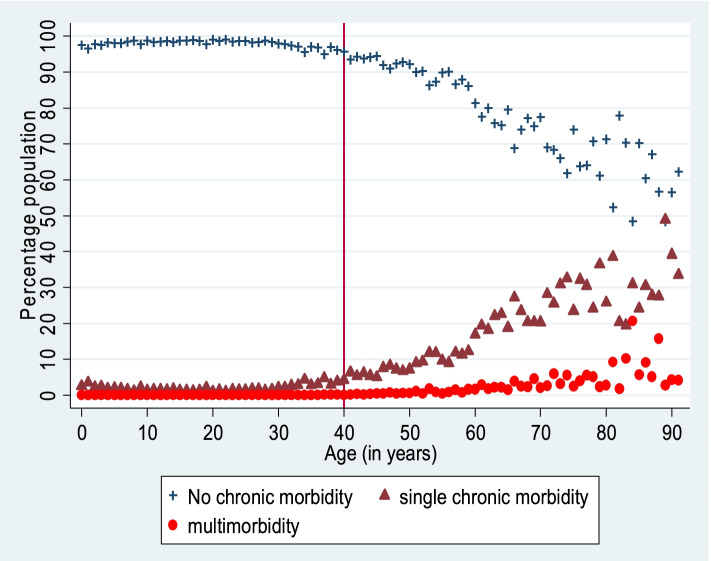
Percentage of population with no, single and multi-NCDs Source: Authors’ estimates using SCH 2017–18

Our analysis suggests that among men of age 40 years and above, 19.7% reported at least one NCD and 4.52% reported two or more co-existent NCDs whereas, among women of the same age group, 21.7% reported at least one NCD and 4.6% reported two or more co-existent NCDs (Table 1). Although all age groups reported the presence of NCD and multimorbidity, the prevalence of multimorbidity increased with increasing age, particularly after the age of 40 years (Fig. 1).

**Table1:** Percentage population reporting nil, single and more than one morbidity in age group 40 years and above, by men and women (15 days’ recall reference)

	Men				Women			
	Number of chronic diseases				Number of chronic diseases			
Age	0	1	2	> 2	0	1	2	> 2
40–44	95.45	4.32	0.23	0.01	91.96	7.53	0.48	0.03
45–49	93.33	5.79	0.73	0.15	89.24	9.19	1.36	0.21
50–54	90.02	8.83	0.91	0.24	86.38	11.1	1.95	0.57
55–59	88.65	10	1.12	0.22	83.23	13.73	2.39	0.66
60–64	74.63	20.58	3.31	1.49	75.78	18.84	4.26	1.12
65–69	74.42	20.14	4.11	1.33	71.28	22.66	3.57	2.48
70–79	66.36	24.91	6.18	2.55	67.92	24.17	4.17	3.75
80&above	59.65	26.83	8.66	4.85	60.62	29.43	6.03	3.92
40&above	80.31	15.18	3.16	1.36	78.30	17.08	3.03	1.59

Source: Authors’ estimates using SCH 2017–18

In Table 2 we present prevalence sex- and age-stratified analysis (for the individuals of age ≥ 40 years) of the most common NCDs. The most common NCDs among men and women were diabetes followed by hypertension, respiratory and cardiovascular conditions. More than 2.4% of men and 2.9% of women in the age group of 40–59 reported diabetes while the prevalence was approximately 9% both among men and women of age 60 years and above. Hypertension was reported by approximately 2% of men and 3% of women in the age group of 40–59 which increased to more than 10% and 11% among men and women respectively aged 60 years and above.

**Table 2:** Percentage of men and women in age groups of 40–59 and 60 + reporting different non-communicable health conditions (15 days recall reference)

	Age 40–59		Age 60 & above	
	Men	Women	Men	Women
	Mean (95% CI)	Mean (95% CI)	Mean (95% CI)	Mean (95% CI)
Cancers	0.08 (0.06–0.10)	0.11 (0.08–0.14)	0.28 (0.22–0.35)	0.20 (0.14–0.26)
Diabetes	2.40 (2.28–2.54)	2.88 (2.75–3.02)	8.70 (8.34–9.06)	8.53 (8.16–8.90)
Mental disorders	0.10 (0.07–0.12)	0.12 (0.09- 0.14)	0.27 (0.20–0.33)	0.11 (0.07–0.16)
Epilepsy	0.03 (0.01–0.04)	0.01 (0.00–0.02)	0.11 (0.07–0.16)	0.08 (0.04–0.11)
Other neurological disorders	0.31 (0.27–0.36)	0.40 (0.35–0.45)	1.74 (1.57–1.90)	1.38 (1.23 -1.53)
Hypertension	1.94 (1.83–2.05)	3.09 (2.95–3.23)	10.22 (9.83–10.61)	11.34 (10.92–11.76)
Cardiovascular disorders	0.76 (0.69–0.83)	0.68 (0.61–0.74)	4.04 (3.78–4.29)	2.74 (2.52–2.95)
Respiratory disorders	0.62 (0.56–0.69)	1.16 (1.07–1.24)	3.26 (3.03–3.49)	2.66 (2.45–2.88)
Musculoskeletal disorders	0.89 (0.82–0.97)	2.25 (2.14–2.37)	4.49 (4.22–4.75)	6.31 (5.99–6.63)
Genitourinary disorders	0.33 (0.28–0.37)	0.44 (0.39–0.49)	1.08 (0.94–1.21)	0.47 (0.38–0.56)
Injury	0.72 (0.65–0.79)	0.38 (0.33–0.43)	0.94 (0.82–1.07)	0.73 (0.62–0.84)
# of observation	59,073	59,636	23,042	21,985

Note: Numbers in parenthesis are confidence intervals at 95% level

Source: Authors’ estimates using SCH 2017–18

We also identified the most commonly associated NCDs in the case of multimorbidity by cross-classifying NCDs at individual reporting levels (see Additional file 1: Appendix Table A-III). The most frequently associated NCDs are hypertension and diabetes followed by diabetes and cardiovascular. For example, among all persons reporting hypertension, approximately 73.5% reported hypertension as standalone morbidity, whereas approximately 15% reported diabetes and 4% reported cardiovascular disease in addition to hypertension. Similarly, among all persons reporting diabetes approximately 77.4% reported diabetes alone, whereas around 16.1% reported hypertension and 3.3% reported cardiovascular disease in addition to diabetes. Cancers and cardiovascular are usually high expenditure conditions and both reflect high comorbidity with diabetes and hypertension. Cardiovascular also reflects high comorbidity with respiratory and musculoskeletal conditions.

### Cost of treating NCDs and multimorbidity

**Fig. 2:**
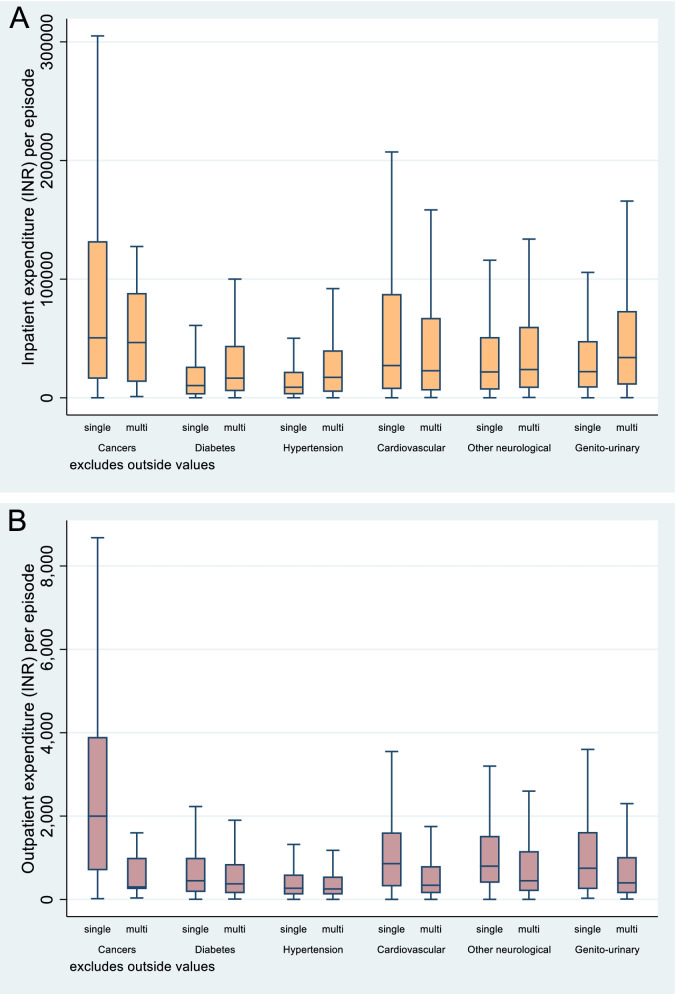
**A** Box plot of per episode median OOP expenditures (INR ‘000’) on the treatment of selected NCDs for outpatient care with and without multimorbidity in the matched sample. **B** Box plot of per episode median OOP expenditures (INR ‘000’) on the treatment of selected NCDs for inpatient care with and without multimorbidity in the matched sample Source: Authors’ estimates using SCH 2017–18

We present results on per episode mean OOP expenditure on selected NCDs in the presence and absence of multimorbidity separately for outpatient and inpatient using the matched sample (Table 3). We also present results with and without including deceased individuals because of the selected NCDs. The results reflect that for hospitalization cases, mean OOP expenditures are mostly significantly higher for the NCD conditions in the presence of multimorbidity as compared with single morbidity, except for cancers and cardiovascular conditions. For the hospital-based treatment of cancers, the mean OOP expenditures are INR 121 thousand (USD 1,900) and INR 74 thousand (USD 1,160) in the absence and presence of multimorbidity respectively. For cardiovascular conditions, the reduction in OOP expenditure is from INR 70 thousand (USD 1,090) in case of single morbidity to INR 60 thousand (USD 938) in the presence of multimorbidity. Moreover, significantly lower mean values of mean OOP expenditure on cancers and cardiovascular conditions in the presence of multimorbidity are mainly triggered by smaller OOP expenditures in the high expenditure brackets (say above-median expenditure levels) as compared with single morbidity (Fig. 2A). For other NCDs as presented in Fig. 2A, expenditure above the median level is significantly higher in the presence of multimorbidity compared to that for a single condition.

**Table 3:** Per episode mean outpatient (15 days) and inpatient (365 days) expenditure (INR@) for various NCD’s with and without multimorbidity in the matched sample

		Excluding deaths				Including deaths			
		Outpatient		hospitalization		Outpatient		hospitalization	
		N	OOP (INR)	N	OOP (INR)	N	OOP (INR)	N	OOP (INR)
Cancer	Single morbidity	260	4,349	627	1,20,271	261	4,349	804	120,726
	Multimorbidity	95	1,837	87	72,091	96	2,374	96	74,200
	Difference$		-2,511**		-48,180*		-1,975*		-46,526*
Diabetes	Single morbidity	4,068	802	1,344	26,600	4,068	802	1,381	26,622
	Multimorbidity	3,380	639	2,027	48,422	3,381	655	2,032	48,393
	Difference$		-163***		21,822***		-147***		21,772***
Hypertension	Single morbidity	3,746	538	1,206	20,358	3,746	538	1,245	20,397
	Multimorbidity	3,774	559	2,150	43,878	3,774	558	2,153	43,876
	Difference$		21		23,520***		20		23,479***
Cardiovascular	Single morbidity	1,228	1,417	2,672	69,792	1,229	1,417	2,904	69,587
	Multimorbidity	1,514	750	1,174	59,474	1,514	750	1,181	59,821
	Difference$		-667***		-10,318***		-666***		-9,766**
Neurologic disorders	Single morbidity	586	1,441	1,220	48,216	586	1,441	1,353	48,226
	Multimorbidity	532	937	517	54,946	534	935	523	55,170
	Difference$		-504***		6,730		-507***		6,944
Genitourinary disorders	Single morbidity	304	1,841	1,708	40,264	304	1,841	1,766	40,483
	Multimorbidity	537	1,126	541	60,202	537	1,126	543	60,447
	Difference$		-715***		19,938***		-715***		19,964***
Any NCD	Single morbidity	15,757	880	17,127	39,662	15,759	880	18,023	39,900
	Multimorbidity	5,944	712	3,954	47,943	5,947	720	3,976	48,156
	Difference$		-168***		8,281***		-160***		8,256***

Notes: @ 1 US $-63.8 INR as of 1^st^ January 2018; N is number of episodes; $ = ( multi-single)

Source: Authors’ estimates using SCH 2017–18

^*^p < 0.01

^**^p < 0.05

^***^p < 0.005

In the case of outpatient care, the mean OOP expenditure is invariably lower for the treatment of the selected NCDs in the presence of multimorbidity as compared with no multimorbidity. However, the differences are much higher in the case of treatment of cancers, cardiovascular and genitourinary conditions in the presence of multimorbidity (see also Fig. 2B). Figure 2B again indicates that for the treatment of cancers and cardiovascular in the presence of multimorbidity, it is the high bracket OOP expenditure which declines significantly. Including or excluding deceased individuals in the sample of any NCD does not make difference to the overall trend, except in the case of cancer and cardiovascular where excluding deceased individuals increases the differences in the OOP expenditure concerning the single morbidity case. Additional file 1: Appendix Table A-VII presents more detailed results.

**Table 4:** Mean per capita monthly OOP expenditure, OOP expenditure as a share of household total consumption expenditure, and percentage of households reporting OOP expenditure being higher to 10% and 20% of households’ total consumption expenditure

		Mean per capita monthly OOP expenditure (INR)	Mean OOPE share to total consumption expenditure	% households reporting catastrophic OOPE at 10% threshold)^a^	% households reporting c atastrophic OOPE at 20% threshold^b^	# Observations
Acute illness		330 (321.74- 338.81)	15.62 (15.25- 15.99)	39.763 (39.762–39.764)	20.184 (20.183–20.185)	34,437
Any NCD		528 (512.59- 543.64)	21.49 (20.82- 22.15)	46.675 (46.674–46.676)	27.896 (27.895–27.897)	36,026
Multimorbidity		1203 (1120.53- 1286.69)	33.13 (31.21- 35.04)	63.360 (63.359–63.361)	40.872 (40.871–40.873)	3,953
Cancer	Single	2326 (2006.58- 2646.74)	84.28 (74.75- 93.80)	88.947 (88.946–88.948)	70.399 (70.398–70.400)	897
	Multimorbidity	2116 (853.36- 3380.33)	61.90 (41.56- 82.24)	80.106 (80.105–80.108)	77.869 (77.868–77.871)	80
Diabetes	Single	591 (558.32- 625.25)	20.04 (18.92- 21.15)	46.539 (46.538–46.540)	27.973 (27.972–27.974)	4,417
	Multimorbidity	1271 (1134.47- 1407.72)	31.10 (28.19- 34.00)	55.386 (55.385–55.387)	38.095 (38.094–38.096)	1,831
Cardiovascular	Single	948 (883.96- 1012.41)	31.96 (30.16- 33.75)	61.791 (61.790–61.792)	43.049 (43.048–43.050)	3,584
	Multimorbidity	1831 (1597.25- 2065.75)	46.98 (42.56- 51.40)	79.800 (79.799–79.801)	54.451 (54.450–54.452)	930
Respiratory	Single	516 (470.24- 563.07)	24.25 (22.48- 26.00)	54.499 (54.498–54.500)	34.068 (34.067–34.069)	2,316
	Multimorbidity	1137 (914.17- 1361.08)	35.52 (30.76- 40.26)	67.214 (67.213–67.216)	45.107 (45.106–45.109)	474

^a^at 10% cut-off of total household consumption expenditure

^b^at 20% cut-off of household consumption expenditure

Table 4 presents a few dimensions of the burden of OOP expenditures related to NCDs at the household level in a full sample of households reporting at least one NCD. This includes indicators such as the mean per capita monthly OOP expenditure (INR), the share of OOP expenditure to household total consumption expenditure and the percentage of households incurring OOP expenditure of more than 10% and 20% of total household consumption expenditure separately for each NCDs. Our analysis suggests that around OOP expenditure as a share of total household expenditure was 21.5% and 33% without and with the presence of multimorbidity respectively. Accordingly, 46.7% of households had incurred catastrophic spending (10% threshold) because of any NCD when it presented as a standalone disease while under multimorbidity scenarios, households reporting catastrophic spending rose to 63.3%.

Furthermore, our analysis also suggests that for cancers and cardiovascular the proportions of households reporting catastrophic expenditure (10% threshold) were more than 88% and 62% respectively in the absence of multimorbidity. With multimorbidity, the proportion marginally declines (80%) for cancers but further increases to more than 79% for cardiovascular. Even at the 20% threshold level, the percentage of households reporting catastrophic OOP expenditure is as high as 77% for cancers and 54% for cardiovascular.

## Discussions

This study, using nationally representative data, presents the prevalence of different NCDs and OOP expenditures on their treatment, in two scenarios (with and without multimorbidity) among the 40 + years population. The burden of OOP expenditure on account of NCDs and multimorbidity incurred by households has been estimated in terms of OOP expenditure as a share of total household consumption expenditure and catastrophic OOP expenditure. To the best of our knowledge, ours is the first study to report the OOP treatment cost for selected NCDs in the standalone scenario and in the presence of competing NCDs (multimorbidity scenario) and also in terms of catastrophic OOP expenditure of selected NCDs on households in the presence and absence of multimorbidity.

Our estimates suggest that in the case of hospitalization, OOP expenditure on high expenditure conditions like cancer and CVD remain underfinanced by approximately 39% and 14% respectively in the presence of multimorbidity compared with a situation when cancers and cardiovascular are reported without multimorbidity. Outpatient care for these conditions too is underfinanced in a range of 45–50% in the presence of multimorbidity in comparison to standalone diseases. This essentially implies that disease conditions with a high propensity to cause catastrophic expenditure at the household level are inadequately financed/treated in the presence of multimorbidity. Various explanations are possible for this. First, although OOP expenditure on treatment of multimorbidity, in general, is higher as compared with that for single NCD, the households are unable to fully fund the treatment of cancers and cardiovascular, which involve high expenditure, in the presence of multimorbidity as they have to allocate resources for the treatment of competing conditions within the given budget constraints. We estimated that in the case of cancers and cardiovascular in the presence of multimorbidity, the percentage of households reporting OOP expenditure being catastrophic is much higher as compared with other NCDs. Second, it is possible that households are accessing treatment in institutions where the treatment cost of cancers and cardiovascular is less such as in the public sector and charitable institutions, or fully or partially financing treatment through insurance for the leading disease but not for competing conditions. Third, in the presence of multimorbidity, the severity of disease for cancers and cardiovascular illness is such that households lose any hope for the survival of the patient and stop spending on formal care and move to home care. Certainly, the last explanation needs deeper studies to fully understand the factors behind underfinancing of high expenditure diseases in the presence of multimorbidity.

Like earlier studies from India and South Asia [17, 25], our analysis also suggests a high proportion of co-existence of NCDs like diabetes, and hypertension, diabetes and cardiovascular disease, cardiovascular disease and hypertension. The co-existence of diabetes, hypertension and cardiovascular diseases has been documented widely and has been explained through commonality in their risk factors [26]. Literature suggests that strategies for the prevention, control and management of multimorbidity should take into account the interaction effects of co-existent NCDs [27], unfortunately, there is a fragmentation of primary care and the absence of referral pathways in the LMICs [28]. It is widely recognized that multimorbidity is associated with higher healthcare utilization rates and increased healthcare expenditure [12, 17, 29]. However, we found no research evidence on the financial implications of co-existing NCDs in terms of OOP expenditures between competing NCDs and their catastrophic implications at the household level. The key contribution of our study is to highlight households’ inability to maintain requisite healthcare expenditure in presence of competing priority NCDs (multimorbidity), especially in cancer and cardiovascular diseases related to hospitalization. This is also reflected in the high proportion of catastrophic expenditures in households that reported cancer and cardiovascular diseases and multimorbidity.

It is recognized that cancer often requires relatively expensive, and complex, treatment for extended periods, which may lead to household impoverishment, treatment abandonment, and poor outcomes, if the disease is detected at a later stage [30]. This is also true to some extent for cardiovascular diseases, which require medicines for a lifetime and may require expensive cardiac interventions such as angiography and angioplasty at the later stages of illness. Such situations may lead to sharing of expenditure on competing NCDs and/or catastrophic expenditure and may lead to distress sale of assets and borrowing. Literature also suggests that cancer-affected households had to rely on borrowing or asset sales for financing treatment [15]. Mehlis et al. reported that 40% of the cancer patients saved money by cutting back on nutrition, living, and medication that is not reimbursed by their health insurance [31]. Mahal et al. have reported that out-of-pocket health expenditures are significantly higher in households with cancer compared to control households, both in inpatient and outpatient settings [16] while Engelgau et al. reported that odds of catastrophic hospitalization expenditure for cancer were nearly 170% greater than that due to the communicable diseases [13]. Our analysis also suggests that around 46.7% of households had incurred catastrophic spending (10% threshold) because of any NCD, which increased to around 63.3% in presence of multimorbidity as compared to around 39.4% for any communicable disease.

However, we also observed that OOP expenditure on NCDs such as diabetes and hypertension was not compromised in presence of another NCD and were higher (may be due to increased risk or severe symptoms) in multimorbidity situation both in the outpatient setting as well as during hospitalization. These observations were consistent with the previous research. A systematic review indicated that multimorbidity was associated with higher OOP expenditures on medicines and with an increase in the number of NCDs from 0 to 1, 2, and ≥ 3, the annual OOP expenditure on medicines increased by an average of 2.7 times, 5.2 times and 10.1 times, respectively [32] which was especially true for NCDs like diabetes and hypertension that require lifelong medication for disease management [33].

Our estimates of self-reported prevalence and multimorbidity from NSSO survey data are relatively lower as compared to previously published estimates from India [8, 17]. This could be due to differences in survey design, study population, method of data collection and number and type of diseases included in the studies [34]. While there are several key strengths of the NSSO survey data—national level representativeness, large sample size and robust estimates on household-level healthcare expenditure [9], under-reporting of morbidity is one of the limitations of NSSO. However, this doesn’t affect our estimates on the OOP expenditure of NCDs and their catastrophic implications on households as we estimated per episode cost of treatment and cost of treatment as a share of household total consumption expenditure. There are a few other limitations of our study. For instance, there is a potential that conditions like cancers and cardiovascular just dominate any other competing conditions in terms of patient recall/reporting in the case of multimorbidity and in our analysis we might have classified many multimorbid episodes under ‘single’ because of the recall bias. Such cases can potentially bias our estimates on OOP expenditure downwardly for one or another NCD in the presence of multimorbidity. However, since in the case of cancers and cardiovascular, diabetes and hypertension are the most frequently competing conditions requiring regular medication, we believe that the probability of such biases is smaller in the case of cancers and cardiovascular episodes. Yet another limitation could be complexities of multimorbidity are not well captured in the data we have used. It is quite possible that the complexities of multimorbidity may lead to differential expenditure patterns on treatment. However, we believe that the rigorous matching method used in this study may have reduced such biases to some extent if not all. Furthermore, the NSSO survey relies on self-reported multimorbidity, hence recall bias cannot be ruled out. Similarly, recall bias for expenditure estimates related to inpatient care is also possible because of the one-year recall period for hospitalization expenditures. However, outpatient expenditure estimates remain robust since the recall period is only 15 days.

## Conclusions

Our analysis and resulting estimates demonstrate that irrespective of the number of co-existing NCDs, households face catastrophic OOP expenditures. In addition, in presence of two or more co-existent NCDs (multimorbidity), the number of households reporting catastrophic OOP expenditure increased. Even more important finding pertains to the reduction in OOP expenditure in cancer and cardiovascular related hospitalization in presence of competing NCDs (multimorbidity) which reflects household budgetary constraints. In this context, it is important to design and implement such financial risk protection measures that explicitly reduce the burden of household OOP expenditure but also reward individuals to reduce risk factors for NCDs. Existing Prime Minister *Jan Aarogya Yojana* (PM-JAY) may consider developing an integrated financial package for multimorbidity. In addition, wider dissemination of information on population-based screening program programs for early detection of hypertension, diabetes and cancer should be ensured to improve their uptake and utilisation. The Health and Wellness Centre Schemes under *Ayushman Bharat* may be utilized to develop integrated early disease detection and treatment and care pathways, especially for cancers and cardiovascular-related multimorbidity.

## Supplementary Information

**Additional file 1: Appendix Table A-I.** Question asked in NSSO 75th Round related to morbidity and utilisation of healthcare at individual and episode level along with recall period for respective questions. **Appendix Table A-II.** Socio-economic personal and household attributes of sample individuals. **Appendix Table A-III.** Percentage of ailing persons with different diseases reporting comorbidity with other diseases. **Appendix Table A-IV.** Categorisation and sample size of various variables used in CEM. **Appendix Table A-V.** Measures of imbalance in the sample before and after CEM. Appendix Table A-VI: Balancing property: Mean of matched and unmatched variables across treatment and control and % bias and % reduction in bias in variables after matching (PSM sensitivity Analysis). **Appendix Table A-VII.** Mean monthly total expenditure for various NCD’s matched (CEM) death cases only.

## Data Availability

The datasets were derived from sources in the public domain: NSSO: Social Consumption and Health 75^th^ round and can be downloaded upon registration and filling in basic details at https://www.mospi.gov.in/web/mospi/download-tables-data/-/reports/view/templateTwo/16202?q=TBDCAT.
