# Supplementary material for: Multimorbidity, healthcare use and catastrophic health expenditure by households in India: a cross-section analysis of self-reported morbidity from national sample survey data 2017–18
Source: BMC Health Serv Res. 2022 Sep 12;22:1151. doi: 10.1186/s12913-022-08509-x (PMC9469515; doi:10.1186/s12913-022-08509-x)
Supplement: Supplementary file 1 — Additional file 1: Appendix Table A-I. Question asked in NSSO 75th Round related to morbidity and utilisation of healthcare at individual and episode level along with recall period for respective questions. Appendix Table A-II. Socio-economic personal and household attributes of sample individuals. Appendix Table A-III. Percentage of ailing persons with different diseases reporting comorbidity with other diseases. Appendix Table A-IV. Categorisation and sample size of various variables used in CEM. Appendix Table A-V. Measures of imbalance in the sample before and after CEM. Appendix Table A-VI: Balancing property: Mean of matched and unmatched variables across treatment and control and % bias and % reduction in bias in variables after matching (PSM sensitivity Analysis). Appendix Table A-VII. Mean monthly total expenditure for various NCD’s matched (CEM) death cases only. [file 12913_2022_8509_MOESM1_ESM.docx]

Appendix Table A-I: Question asked in NSSO 75^th^ Round related to morbidity and utilisation of healthcare at individual and episode level along with recall period for respective questions

|  | **Question in the survey** |  | **Recall period** |
| --- | --- | --- | --- |
| **Individual level** |  |  |  |
|  | Whether hospitalised (yes-1, no-2) |  | 365 days |
|  |  | if yes, number of times hospitalised | 366 days |
|  | Whether suffering from any chronic ailment (yes-1, no-2) |  | 15 days |
|  | Whether suffered/suffering from any other ailment (besides chronic ailment) |  |  |
|  |  | anytime during last 15 days (yes-1, no-2) | 15 days |
|  |  | on the date before the date of survey (yes-1, no-2) | 15 days |
| **Episode level -- hospitalisation** |  |  |  |
|  | Nature of ailment | classified by 60+ different codes | 365 days |
|  | Expenditure for treatment during stay at hospital (in whole number of Rs.) | classified by 8 different items* | 365 days |
| **Episode level – non-hospitalised cases** |  |  |  |
|  | Nature of ailment | classified by 60+ different codes | 15 days |
|  | Whether chronic (yes-1, no-2) |  |  |
|  | Medical/non-medical expenditure for treatment (in whole number of Rs.) | classified by 7 different items** | 15 days |

**Notes**: * includes items of expenditure namely – i. package component (Rs.), ii. doctor’s/ surgeon’s fee (hospital staff/ other specialists), iii. Medicines, iv. diagnostic tests, v. bed charges, vi. other medical expenses (attendant charges, physiotherapy, personal medical appliances, blood, oxygen, etc. vii. transport for patient (Rs.), viii. other non-medical expenses incurred by the household (registration fee, food, transport for others, expenditure on escort, lodging charges if any, etc.) (Rs.); ** * includes items of expenditure namely – i. doctor’s/ surgeon’s fee (hospital staff/ other specialists), ii. medicines: AYUSH (indigenous system), iii. medicines: other than AYUSH (modern system), iv. other medical expenses (attendant charges, physiotherapy, personal medical appliances, blood, oxygen, etc.), v. transport for patient (Rs.), vi. other expenses incurred by the household (registration fee, food, transport for others, expenditure on escort, etc.) (Rs.)

**Source**: NSSO 2017-18, Schedule 25.0

Appendix Table A-II: Socio-economic personal and household attributes of sample individuals

| Variable | Mean | Std. Err. | [95% Conf. Interval] | |
| --- | --- | --- | --- | --- |
| **Gender** |  |  |  |  |
| Male | 0.510 | 0.001 | 0.509 | 0.511 |
| Female | 0.490 | 0.001 | 0.489 | 0.491 |
| **Age group (in years)** |  |  |  |  |
| 0-4 | 0.117 | 0.000 | 0.116 | 0.117 |
| 5-14 | 0.164 | 0.000 | 0.163 | 0.165 |
| 15-29 | 0.276 | 0.001 | 0.275 | 0.277 |
| 30-44 | 0.209 | 0.001 | 0.208 | 0.210 |
| 45-59 | 0.158 | 0.000 | 0.157 | 0.159 |
| 60-69 | 0.050 | 0.000 | 0.049 | 0.051 |
| 70&above | 0.027 | 0.000 | 0.027 | 0.027 |
| **Levels of education** |  |  |  |  |
| Illiterate | 0.265 | 0.001 | 0.264 | 0.266 |
| Below Primary | 0.152 | 0.000 | 0.151 | 0.153 |
| Up to Middle | 0.255 | 0.001 | 0.254 | 0.256 |
| Secondary/higher secondary | 0.232 | 0.001 | 0.231 | 0.233 |
| Graduate and above | 0.095 | 0.000 | 0.094 | 0.096 |
| **Area of residence** |  |  |  |  |
| Rural | 0.587 | 0.001 | 0.586 | 0.588 |
| Urban | 0.413 | 0.001 | 0.412 | 0.414 |
| **Religion** |  |  |  |  |
| Hindu | 0.743 | 0.001 | 0.742 | 0.744 |
| Muslim | 0.150 | 0.000 | 0.149 | 0.150 |
| Others | 0.107 | 0.000 | 0.107 | 0.108 |
| **Social group** |  |  |  |  |
| Scheduled tribe | 0.136 | 0.000 | 0.135 | 0.136 |
| Scheduled caste | 0.169 | 0.001 | 0.168 | 0.170 |
| Other backward classes | 0.401 | 0.001 | 0.400 | 0.403 |
| Others | 0.294 | 0.001 | 0.292 | 0.295 |
| **Main source of livelihood** |  |  |  |  |
| Self-employed in agriculture | 0.244 | 0.001 | 0.243 | 0.246 |
| Self-employed in non-agriculture | 0.278 | 0.001 | 0.277 | 0.279 |
| Regular salary work | 0.223 | 0.001 | 0.222 | 0.224 |
| Casual salary work | 0.212 | 0.001 | 0.211 | 0.213 |
| Others | 0.043 | 0.000 | 0.042 | 0.043 |
| **source of drinking water** |  |  |  |  |
| Safe source | 0.936 | 0.000 | 0.935 | 0.936 |
| Unsafe source | 0.064 | 0.000 | 0.064 | 0.065 |
| **Source of sanitation** |  |  |  |  |
| Safe | 0.762 | 0.001 | 0.761 | 0.763 |
| Unsafe | 0.238 | 0.001 | 0.237 | 0.239 |
| **Source of fuel** |  |  |  |  |
| Non solid | 0.623 | 0.001 | 0.621 | 0.624 |
| Solid | 0.371 | 0.001 | 0.370 | 0.372 |
| Number of observation | 555,352 |  |  |  |

Source: Authors’ estimates using SCH 2017-18

Appendix Table A-III: Percentage of ailing persons with different diseases reporting comorbidity with other diseases

|  | Cancers | Diabetes | Mental  disorders | Neurol  ogical  disorders | Hyper  tension | Cardio  vascular disorders | Respi  ratory  disorders | Musculo  skeletal  disorders | Genito  urinary  disorders | Injuries |
| --- | --- | --- | --- | --- | --- | --- | --- | --- | --- | --- |
| Cancers | **0.881** | 0.048 | 0.000 | 0.006 | 0.027 | 0.011 | 0.004 | 0.003 | 0.003 | 0.010 |
| Diabetes | 0.002 | **0.774** | 0.001 | 0.009 | 0.161 | 0.033 | 0.014 | 0.023 | 0.010 | 0.009 |
| Mental  disorders | 0 | 0.037 | **0.808** | 0.008 | 0.048 | 0.012 | 0.010 | 0.011 | 0.006 | 0.009 |
| Neurol  ogical  disorders | 0.001 | 0.036 | 0.001 | **0.842** | 0.089 | 0.014 | 0.008 | 0.013 | 0.007 | 0.005 |
| Hyper  tension | 0.001 | 0.150 | 0.002 | 0.020 | **0.735** | 0.040 | 0.017 | 0.036 | 0.008 | 0.008 |
| Cardio  vascular disorders | 0.002 | 0.099 | 0.001 | 0.011 | 0.127 | **0.766** | 0.022 | 0.020 | 0.012 | 0.012 |
| Respi  ratory  disorders | 0.000 | 0.037 | 0.001 | 0.005 | 0.049 | 0.019 | **0.884** | 0.025 | 0.005 | 0.004 |
| Musculo  skeletal  disorders | 0.000 | 0.034 | 0.001 | 0.005 | 0.057 | 0.010 | 0.014 | **0.886** | 0.004 | 0.007 |
| Genito urinary  disorders | 0.001 | 0.081 | 0.002 | 0.014 | 0.067 | 0.031 | 0.013 | 0.020 | **0.822** | 0.005 |
| Injuries | 0.002 | 0.052 | 0.002 | 0.006 | 0.050 | 0.023 | 0.009 | 0.026 | 0.004 | **0.840** |

Source: Authors’ estimates using SCH 2017-18

Appendix Table A-IV: Categorisation and sample size of various variables used in CEM

| **Geographical Regions** | Number of Individuals in sample | Percentage |
| --- | --- | --- |
| North | 5,265 | 13.86 |
| West | 6,299 | 16.58 |
| East | 7,103 | 18.69 |
| South | 12,727 | 33.5 |
| Central | 4,127 | 10.86 |
| North East | 2,475 | 6.51 |
| **Sector** |  |  |
| Rural | 18,936 | 49.84 |
| Urban | 19,060 | 50.16 |
| **Religion** |  |  |
| Hindu | 28,382 | 74.7 |
| minority | 9,614 | 25.3 |
| **Social Group** |  |  |
| SC/ST | 7,965 | 20.96 |
| OBC | 14,911 | 39.24 |
| Others | 15,120 | 39.79 |
| **Main source of livelihood** |  |  |
| Self-employed | 17,477 | 46 |
| Salaried | 9,452 | 24.88 |
| Casual labour | 11,067 | 29.13 |
| **Level of Education** |  |  |
| Illiterate | 14,078 | 37.05 |
| Upto higher secondary | 20,545 | 54.07 |
| graduate and above | 3,373 | 8.88 |
| **Insurance Status** |  |  |
| Insured | 10,347 | 27.23 |
| Not-Insured | 27,649 | 72.77 |
| **Multimorbidity** |  |  |
| single | 31,252 | 82.25 |
| multi | 6,744 | 17.75 |
| Total | 37,996 | 100 |

Appendix Table A-V: Measures of imbalance in the sample before and after CEM

|  | UNMATCHED | | MATCHED | |
| --- | --- | --- | --- | --- |
| Multivariate L1 imbalance: | 0.964524 |  | 0.956332 |  |
| Univariate imbalance: |  |  |  |  |
|  | L1 imbalance | Difference in means | L1 imbalance | Difference in means |
| geographical region | 0.29765 | 0.12298 | 0.02388 | 0.02388 |
| sector | 0.07716 | 0.07716 | 0.06034 | 0.06034 |
| mpce | 0.2106 | 863.79 | 0.15287 | 579.03 |
| religion | 0.07456 | 0.07456 | 0.08026 | 0.08026 |
| social group | 0.07368 | 0.13504 | 0.00726 | 0.00726 |
| household type | 0.09231 | 0.16112 | 0.03238 | 0.03238 |
| age | 0.18082 | 4.2552 | 0.10879 | 1.2996 |
| education | 0.13949 | 0.15667 | 0.11546 | 0.11546 |
| safe_latrine | 0.07925 | -0.07925 | 0.05934 | -0.05934 |
| safe_water | 0.13954 | 0.13954 | 0.0753 | 0.0753 |
| safe_garb | 0.0007 | 0.0007 | 0.02003 | 0.02003 |
| safe_energy | 0.02451 | -0.02451 | 0.03154 | 0.03154 |
| insurance | 0.16901 | -0.16901 | 0.09174 | -0.09174 |

Source: Authors’ estimates using SCH 2017-18

Appendix Table A-VI: Balancing property: Mean of matched and unmatched variables across treatment and control and % bias and % reduction in bias in variables after matching (PSM sensitivity Analysis)

| variables |  | Mean | |  | %reduction | t-test | |
| --- | --- | --- | --- | --- | --- | --- | --- |
|  | Unmatched/ Matched | treated | control | %bias | bias | t | p>t |
|  |  |  |  |  |  |  |  |
| Rural | U | 0.4328 | 0.5149 | -16.5 |  | -7.54 | 0 |
|  | M | 0.4328 | 0.4309 | 0.4 | 97.7 | 0.17 | 0.866 |
| Quintile1 | U | 0.08265 | 0.13204 | -16 |  | -7.26 | 0 |
|  | M | 0.08265 | 0.08419 | -0.5 | 96.9 | -0.25 | 0.806 |
| Quintile2 | U | 0.11895 | 0.14948 | -9 |  | -4.08 | 0 |
|  | M | 0.11895 | 0.12096 | -0.6 | 93.4 | -0.27 | 0.785 |
| Quintile3 | U | 0.17533 | 0.19651 | -5.4 |  | -2.49 | 0.013 |
|  | M | 0.17533 | 0.18409 | -2.3 | 58.6 | -1.01 | 0.315 |
| Hindu | U | 0.70984 | 0.75977 | -11.3 |  | -5.19 | 0 |
|  | M | 0.70984 | 0.71098 | -0.3 | 97.7 | -0.11 | 0.911 |
| Muslim | U | 0.15139 | 0.14529 | 1.7 |  | 0.79 | 0.432 |
|  | M | 0.15139 | 0.1514 | 0 | 99.9 | 0 | 0.999 |
| ST | U | 0.0363 | 0.05807 | -10.3 |  | -4.66 | 0 |
|  | M | 0.0363 | 0.03854 | -1.1 | 89.7 | -0.52 | 0.604 |
| SC | U | 0.12822 | 0.14926 | -6.1 |  | -2.78 | 0.005 |
|  | M | 0.12822 | 0.13092 | -0.8 | 87.2 | -0.35 | 0.723 |
| OBC | U | 0.38955 | 0.40406 | -3 |  | -1.36 | 0.175 |
|  | M | 0.38955 | 0.39323 | -0.8 | 74.6 | -0.33 | 0.739 |
| Self-employed in agriculture | U | 0.10324 | 0.1815 | -22.5 |  | -10.21 | 0 |
|  | M | 0.10324 | 0.10418 | -0.3 | 98.8 | -0.14 | 0.892 |
| Self-employed in non-agriculture | U | 0.28038 | 0.25635 | 5.4 |  | 2.48 | 0.013 |
|  | M | 0.28038 | 0.27853 | 0.4 | 92.3 | 0.18 | 0.856 |
| Casual labour | U | 0.16169 | 0.1815 | -5.3 |  | -2.4 | 0.016 |
|  | M | 0.16169 | 0.15882 | 0.8 | 85.5 | 0.34 | 0.731 |
| Age 50-59 | U | 0.25927 | 0.29565 | -8.1 |  | -3.71 | 0 |
|  | M | 0.25927 | 0.26418 | -1.1 | 86.5 | -0.49 | 0.622 |
| Age 60-69 | U | 0.32878 | 0.27799 | 11.1 |  | 5.07 | 0 |
|  | M | 0.32878 | 0.32834 | 0.1 | 99.1 | 0.04 | 0.967 |
| Age 70 & above | U | 0.29197 | 0.21528 | 17.7 |  | 8.12 | 0 |
|  | M | 0.29197 | 0.2885 | 0.8 | 95.5 | 0.34 | 0.736 |
| Illiterate | U | 0.27317 | 0.36851 | -20.5 |  | -9.36 | 0 |
|  | M | 0.27317 | 0.28366 | -2.3 | 89 | -1.03 | 0.302 |
| Below Primary | U | 0.13157 | 0.11769 | 4.2 |  | 1.93 | 0.054 |
|  | M | 0.13157 | 0.13156 | 0 | 100 | 0 | 0.999 |
| Middle | U | 0.276 | 0.24707 | 6.6 |  | 3.02 | 0.003 |
|  | M | 0.276 | 0.27163 | 1 | 84.9 | 0.43 | 0.666 |
| Higher Secondary | U | 0.21035 | 0.18525 | 6.3 |  | 2.89 | 0.004 |
|  | M | 0.21035 | 0.20629 | 1 | 83.8 | 0.44 | 0.659 |
| Safe drinking water | U | 0.02678 | 0.03599 | -5.3 |  | -2.4 | 0.016 |
|  | M | 0.02678 | 0.02611 | 0.4 | 92.7 | 0.18 | 0.854 |
| Safe sanitation | U | 0.08393 | 0.0764 | 2.8 |  | 1.27 | 0.204 |
|  | M | 0.08393 | 0.08086 | 1.1 | 59.2 | 0.49 | 0.622 |
| Safe fuel | U | 0.97013 | 0.97372 | -2.2 |  | -1 | 0.319 |
|  | M | 0.97013 | 0.97324 | -1.9 | 13.6 | -0.82 | 0.41 |
| Insured | U | 0.25618 | 0.18415 | 17.4 |  | 8.01 | 0 |
|  | M | 0.25618 | 0.25591 | 0.1 | 99.6 | 0.03 | 0.978 |
| Ailment 0-5 days | U | 0.02008 | 0.04526 | -14.2 |  | -6.4 | 0 |
|  | M | 0.02008 | 0.02408 | -2.3 | 84.1 | -1.2 | 0.231 |
| Ailment 6-10 days | U | 0.02729 | 0.034 | -3.9 |  | -1.77 | 0.076 |
|  | M | 0.02729 | 0.02935 | -1.2 | 69.3 | -0.55 | 0.584 |
| Hospitalized 0-3 days | U | 0.4225 | 0.41334 | 1.9 |  | 0.85 | 0.395 |
|  | M | 0.4225 | 0.42619 | -0.7 | 59.7 | -0.33 | 0.742 |
| Hospitalized 4-10 days | U | 0.25721 | 0.2866 | -6.6 |  | -3.02 | 0.003 |
|  | M | 0.25721 | 0.2573 | 0 | 99.7 | -0.01 | 0.993 |
| Region- north | U | 0.08574 | 0.14749 | -19.3 |  | -8.76 | 0 |
|  | M | 0.08574 | 0.08787 | -0.7 | 96.5 | -0.33 | 0.738 |
| Region-west | U | 0.15448 | 0.189 | -9.2 |  | -4.18 | 0 |
|  | M | 0.15448 | 0.14765 | 1.8 | 80.2 | 0.84 | 0.401 |
| Region-east | U | 0.16838 | 0.17112 | -0.7 |  | -0.33 | 0.739 |
|  | M | 0.16838 | 0.16841 | 0 | 99.1 | 0 | 0.998 |
| Region-south | U | 0.52497 | 0.3429 | 37.4 |  | 17.12 | 0 |
|  | M | 0.52497 | 0.52433 | 0.1 | 99.6 | 0.06 | 0.955 |
| Region-central | U | 0.05252 | 0.12122 | -24.6 |  | -11.08 | 0 |
|  | M | 0.05252 | 0.05649 | -1.4 | 94.2 | -0.77 | 0.441 |
| Household size | U | 1.3449 | 1.4026 | -10.8 |  | -4.95 | 0 |
|  | M | 1.3449 | 1.3441 | 0.1 | 98.8 | 0.06 | 0.953 |

* if variance ratio outside [0.94; 1.06] for U and [0.94; 1.06] for M

| Sample | Ps R2 | LR chi2 | p>chi2 | MeanBias | MedBias | B | R | %Var |
| --- | --- | --- | --- | --- | --- | --- | --- | --- |
| Unmatched | 0.076 | 885.21 | 0 | 10.1 | 8.1 | 67.1* | 0.84 | 0 |
| Matched | 0.001 | 7.15 | 1 | 0.8 | 0.7 | 6.1 | 1.08 | 0 |

* if B>25%, R outside [0.5; 2]

Source: Authors’ estimates using SCH 2017-18

Appendix Table A-VII: Mean monthly total expenditure for various NCD’s matched (CEM) death cases only

|  |  | UNMATCHED | | | MATCHED | | |
| --- | --- | --- | --- | --- | --- | --- | --- |
|  |  | Mean total monthly expenditure (INR) | %age of monthly household expenditure | N | Mean total monthly expenditure (INR) | %age of monthly household expenditure | N |
|  |  |  |  |  |  |  |  |
| ANY CHRONIC | single | 5476.79 | 43.18011 | 1,102 | 5861.178 | 44.53221 | 414 |
|  | multi | 9096.42 | 72.51151 | 21 | 11750.31 | 80.96827 | 21 |
| CANCER | single | 11130.34 | 94.96812 | 209 | 10108.29 | 92.791 | 20 |
|  | multi | 49101.59 | 475.4423 | 3 | 46898.89 | 610.3977 | 3 |
| DIABETES | single | 3170.72 | 18.08145 | 45 | N.A | N.A | N.A |
|  | multi | 12195.72 | 184.5506 | 5 | N.A | N.A | N.A |
| HYPERTENSION | single | 3425.571 | 32.28007 | 52 | 3425.278 | 75.00608 | 3 |
|  | multi | 1829.711 | 11.62918 | 3 | 3521.944 | 23.96968 | 3 |
| CVD | single | 3254.188 | 23.23174 | 302 | 2717.364 | 13.21575 | 45 |
|  | multi | 8666.806 | 51.81033 | 7 | 10056.5 | 52.69598 | 5 |
| OTHER NEUROLOGIC DISORDERS | single | 3535.977 | 36.34494 | 163 | 7938.765 | 31.54476 | 14 |
|  | multi | 5021.691 | 27.27108 | 5 | 5464.167 | 27.7839 | 3 |
| GENITOURINARY DISORDERS | single | 6246.102 | 66.94147 | 68 | 610.75 | 5.09791 | 5 |
|  | multi | 12169.62 | 90.09141 | 2 | 13500 | 96.42857 | 1 |

Source: Authors’ estimates using SCH 2017-18
